# Analysis Method for the Spatial Layout Equilibrium of Highway Transportation Network Based on Community Detection

**DOI:** 10.3390/s25206366

**Published:** 2025-10-15

**Authors:** Yuanyuan Zhang, Weidong Song, Jinguang Sun, Peng Dai

**Affiliations:** 1 School of Geomatics, Liaoning Technical University, Fuxin 123000, China; zhangyuanyuan@lntu.edu.cn; 2 School of Civil Engineering, Liaoning Technical University, Fuxin 123000, China; 3 School of Electronics and Information Engineering, Liaoning Technical University, Huludao 125105, China; sunjinguang88@163.com; 4 Department of Basic Teaching, Liaoning Technical University, Huludao 125105, China; daipeng@lntu.edu.cn

**Keywords:** highway transportation network, C-Louvain algorithm, network spatial layout equilibrium, demand–supply analysis, Gini coefficient

## Abstract

Analyzing the spatial layout equilibrium of highway transportation networks is essential for optimizing transportation networks, enhancing system efficiency and sustainability. To promote the equitable distribution and management of highway traffic resources, this study introduces a framework for assessing the spatial layout equilibrium of highway networks based on community structure. A new algorithm, named the C-Louvain algorithm, is introduced in this paper to address improving the stability of detection results in unconnected networks. The method first constructs a spatial node-based network, then detects the community structure of the highway network using the C-Louvain algorithm, and identifies key communities of the community structure network through a depth-first search. Network spatial layout imbalance is quantitatively assessed through supply–demand equilibrium analysis based on the Gini coefficient. This methodology is applied to the regional highway network in Shenyang, China. Results indicate that the C-Louvain method is optimal, excelling in accuracy, volatility, and efficiency compared to the classic FN, Leiden, and Louvain algorithms, providing a valuable contribution to the literature on graph clustering and data mining. There are significant differences in the number of communities within different connected components, which reflects the heterogeneity of the network’s structure. By this method, the imbalanced area in the highway transportation network layout is quickly found, and the equitable distribution of traffic resources is quantitatively evaluated. The research results can provide a theoretical basis for managers to make scientific investment decisions for road network construction.

## 1. Introduction

Urban–rural integration has become an important issue in promoting sustainable development and reducing regional imbalances globally [1]. In many developing countries and emerging economies, the contradiction between rapid urbanization and lagging rural development is becoming increasingly prominent. As a key link between urban and rural areas, highway infrastructure plays a crucial role in promoting regional economic integration and improving public service access [2]. However, with the increasing complexity of urban and rural traffic networks, highway trunk lines with different functional grades show significant differences in traffic flow, vehicle types, operating speeds, etc. How to scientifically plan and optimize the spatial layout of highway networks to meet diversified passenger and cargo transportation needs and ensure safe and smooth travel has become a common concern for international academics and policy makers [3]. As the largest developing country in the world, China’s urban and rural highway network construction and optimization practice not only provides important support for the new urbanization and rural revitalization strategy in China but also provides a valuable experience reference for other countries facing similar challenges [4,5,6,7,8].

Equilibrium research is mature in the field of social economy. It is a criterion to judge the rationality or fairness of wealth distribution and resource allocation. Similarly, the space layout equilibrium of a network refers to the significant disparities among sub-regions within a specific area due to inherent factors and varying degrees of advantages. There exists an uncoordinated phenomenon of coexistence of strengths and weaknesses, and a tendency towards polarization in development levels and methods. Taking this area as a system, a reasonable spatial configuration of the entire network is carried out to promote and achieve coordinated economic and social development among sub-regions. Based on the actual economic demands, further consideration is given to the potential demands and comprehensive benefits, such as economic and social aspects of each sub-region, and a reasonable spatial gradient configuration of the networks in each sub-region is conducted [9].

This study proposes that the spatial layout equilibrium of highway traffic networks refers to the degree of equalization of the highway layout, restricted by geographical spatial position and land use in a specific area. Equilibrium is different from average or no difference; that is, the existence of difference is allowed, but the whole should be lower than a certain limit. The key point of the spatial layout equilibrium of highway networks is to provide urban residents with relatively equal travel opportunities, travel efficiency, and service quality [10,11]. From an economic perspective, the highway network is a kind of service resource, and cities are the carriers of this kind of service. A balanced highway network layout is conducive to improving the utilization rate of inter-city highway space, ensuring the full interplay of service functions and the improvement of operational benefits. It is also the objective demand of equalization and inclusive construction of basic public services. Therefore, on the one hand, it is necessary to carry out research on the spatial layout equilibrium of highway networks. On the other hand, it is reasonable and feasible to study it by using economic methods, which will provide more perspectives and bases for highway network layout planning, route construction, site selection, and so on.

Traditional spatial layout equilibrium analysis of networks often starts with a macro perspective, and it is difficult to reveal the local structural characteristics of the network. Community detection, as a complex network analysis method, can identify closely connected node groups in a network, thus revealing spatial characteristics of local supply–demand imbalances [12]. For example, the Louvain algorithm [13] has been widely applied to community partitioning of large-scale networks, and urban road network research also shows that community structure can effectively reflect the aggregation and bottleneck distribution of traffic flow [14,15]. For low-density, decentralized, and differential highway networks, it is important to explore the connection relationship between high-concentration highway groups for analyzing the equalization degree of network layouts. Therefore, the community detection method is introduced in this study to describe highway network structure more precisely, provide structured input for spatial layout equilibrium analysis, and provide a scientific basis for revealing local supply–demand imbalance analysis of highway networks.

While previous studies have focused on Louvain community detection algorithms, they often suffer from the distorted optimization modularity calculation results in disconnected networks, making it impossible to obtain reliable community detection results. In this paper, we introduce a new algorithm, the C-Louvain algorithm, which is the first to tackle improving the stability of detection results in unconnected networks by calculating the modularity and modularity gain of node-degree ordering of connected components. This work represents a valuable contribution to both theory and practice in graph clustering and data mining.

In this paper, we are interested in the quantitative evaluation of the spatial layout equilibrium of highway networks. The proposed method demonstrates the application of complex network theory. The study aims to identify the areas within the highway network where the spatial layout is imbalanced. Our case study focused on the five-level highway network of national, provincial, county, township and village highways in Shenyang, utilizing only structural data and excluding any information about traffic flow.

## 2. Materials and Methods

In this section, we first explain the framework of the method proposed in this paper. Then, the details of each submodule are presented, including the theoretical basis and implementation details.

### 2.1. Methodological Framework

**Figure 1:**
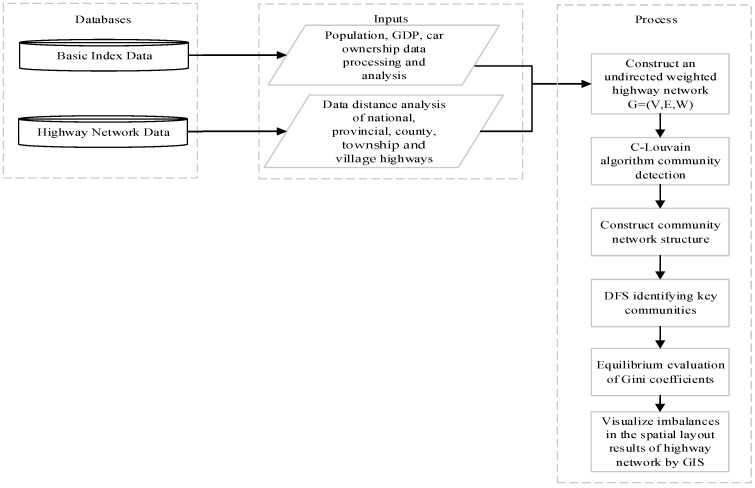
Methodological framework.

Figure 1 shows the methodological framework, and it is divided into three sections: first: required databases, second: inputs from those databases, and third: processes to calculate the spatial layout equilibrium of highway transportation networks. The first database is responsible for storing and managing pertinent data, including indicator data, as well as national, provincial, county township, and village highway network data. The second data input involves data processing and analysis within the database. It is used to calculate the distance in the highway network data. GIS interpolation analysis is conducted on the population, GDP, and car ownership of the highway points. The third calculation process involves using the NetworkX library in Python (3.9.6) to construct the highway transportation network. By using the C-Louvain algorithm to detect community structures, the community structure network can be constructed. Key communities are identified using DFS, while the Gini coefficient analyzes the balance between population, economic, and travel demands, and highway supply. Lastly, we visualize the results using any GIS (10.7) software.

### 2.2. Case Study

According to the research characteristics, actual needs, and engineering applications of this study, a representative five-level highway network in Shenyang City was selected for investigation and statistical analysis in order to quantitatively evaluate the spatial layout equilibrium of highway networks.

Shenyang City was chosen as the case city for the spatial layout equilibrium analysis of highway networks, mainly based on the following considerations: firstly, Shenyang is an important transportation hub in Northeast China and a pilot city of transportation power. Its highway network structure is complex and typical, and many national highways converge here, forming a highway network layout combining radial and loop configurations. Secondly, Shenyang’s economy is developing actively, population and car ownership continue to grow, and traffic demand is strong, which can reflect the main problems faced by the spatial layout equilibrium of highway networks in China. In addition, Shenyang’s highway network data is relatively complete, which is convenient for quantitative analysis and model verification. Therefore, Shenyang’s case not only has local significance, but also provides a reference for other similar cities, with strong representativeness and popularization value.

**Figure 2:**
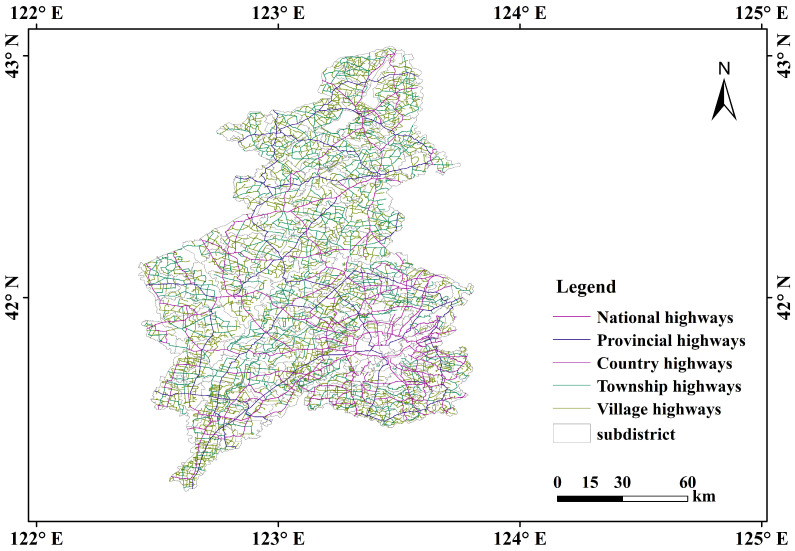
Overview map of the five-level highway network in Shenyang.

Our case study focused on the five-level highway network of national, provincial, county, township, and village highways in Shenyang during 2020, as detailed in Table 1. The dataset includes highway grades, names, numbers, affiliated regions, and geographic locations. Additionally, vector data for county and township administrative regions under Shenyang’s jurisdiction were collected, as depicted in Figure 2.

This study examines five indicators from the 2021 Shenyang Statistical Yearbook: city-level per capita car ownership, county-level per capita GDP and highway mileage, and township-level population and land area. Township population data are derived from sub-district office data in China’s Seventh National Population Census (2020). Each township’s regional GDP is calculated by multiplying the county or district’s per capita GDP by the township population, reflecting regional affluence and economic development. Due to the availability of only city-level car ownership data, township car ownership is estimated by multiplying city per capita car ownership by township population, indicating regional economic development and travel demand.

### 2.3. Highway Network Construction Method

In complex network community structure analysis, the choice of network modeling significantly influences the reliability of detection results and the efficacy of subsequent analyses. Constructing a transportation network is crucial for assessing the network spatial layout equilibrium. The primary construction methods are the primal [15] and dual approaches [16]. The primal approach represents networks with links as edges and nodes as vertices, mapping real geographical data between highway intersections and segments. Conversely, the dual approach abstracts highways into nodes, with intersections mapped as edges representing topological associations [17,18]. This approach is effective for analyzing interactions between highway segments but is limited in assessing large-scale spatial layout structures due to its disregard for highway attributes.

Given the pronounced spatial variations in the geographical distribution of highway grades, the primal approach constructs a spatial node-based highway network. In this highway network, intersections and endpoints of highways are represented as network nodes, while highway segments serve as edges. This network captures the connectivity and spatial positioning of highways.

### 2.4. Description of Community Structure

In graph theory, community structure characterizes a network by its dense intra-community and sparse inter-community connections [19]. A sub-region’s node set forms a community, and the process of identifying these communities is termed community structure detection, or traffic area division [20].

**Figure 3:**
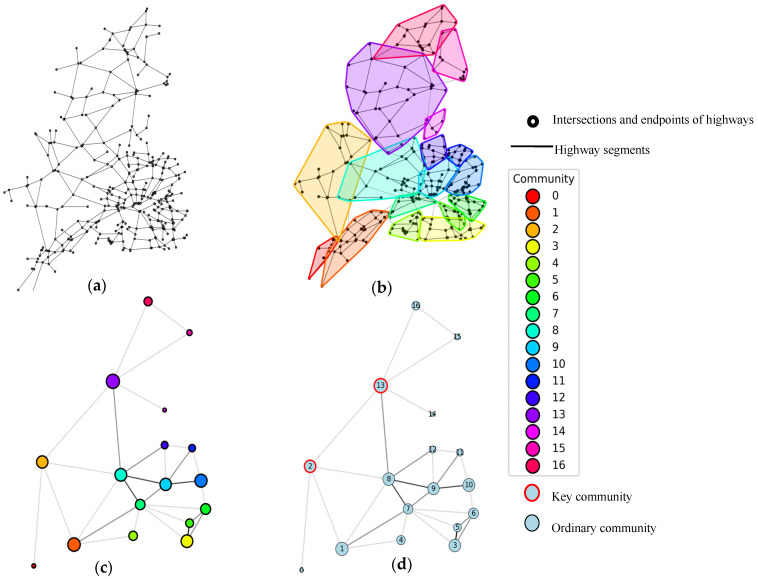
Diagram of community-structured networks. (**a**) Original network structure. (**b**) Community detection results. (**c**) Community structure network. (**d**) Key community identification results.

Figure 3 is diagram of community-structured networks. We selected real case studies of the highway networks in Shenyang, including national, provincial and county highways, to describe the community structure. (a) is the original network structure used to represent the spatial structure of the Shenyang highway network. (b) depicts the community detection results of the highway network. It consists of 16 communities. Within each community, the nodes are densely connected, while the connections between different communities are sparse. This reflects the local structural characteristics of the network. (c) shows the community structure network in order to visualize the size of the communities and the connection between the communities, with the set of nodes in each community mapped to spatial nodes. The node size indicates the size of the community, and the color indicates different communities. (d) presents the key community identification results of the community structure network. These impact network connectivity, reliability, and stability. Identifying and analyzing such key communities can provide insights into the supply–demand balance mechanism of the entire network.

### 2.5. Expression of Connectivity Component

An undirected network G is connected if at least one path exists between any pair of nodes and is disconnected if no path exists. Each disconnected network consists of a number of disjoint connected subgraphs $G=\left\{\left.\bar{G_{1}},\bar{G_{2}},\ldots ,\bar{G_{m}}\right\}\right.$. The connected subgraph that contains the highest number of nodes is called the maximum connected component and represents the maximum range of connectivity in the network structure. A disconnected network can be expressed by a connectivity matrix, so for an undirected network, $G=\left(V,E,W\right)$, $V=\left\{\left.v_{1},v_{2},\ldots ,v_{n}\right\}\right.$, and $P(G)={\left(p_{ij}\right)}_{n\times n}$.

(1) $$p_{ij}=\left\{\begin{array}{l} 1,\begin{array}{cc} & \;v_{i}\;\mathrm{connected}\;\mathrm{to}\;v_{j} \end{array}\\ 0,\begin{array}{cc} & \;v_{i}\;\mathrm{not}\;\mathrm{connected}\;\mathrm{to}\;v_{j} \end{array} \end{array}\right.\begin{array}{cc} & i,j=1,\ldots ,n \end{array}$$

*P*(G) is called the connectivity matrix of G, where V denotes nodes, E denotes edges, W denotes weights, and *n* is the number of network nodes.

The connectivity matrix is a symmetric matrix. For the connectivity components $\bar{G}=\left(\bar{V},\bar{E},\bar{W}\right)$, $\bar{V}=\left\{\left.\bar{v_{1}},\bar{v_{2}},\ldots ,\bar{v_{n}}\right\}\right.$, and $\bar{G}\subset G$, the connectivity component matrix is denoted as

(2) $$\bar{a_{ij}}=\left\{\begin{array}{l} {\bar{\omega }}_{ij},\begin{array}{cc} & \;\;\bar{v_{i}}\;\mathrm{and}\;\bar{v_{j}}\;\mathrm{have}\;\mathrm{side}\;\mathrm{rights} \end{array}\\ 0,\begin{array}{cc} & \;\;\;\;\bar{v_{i}}\;\mathrm{and}\;\bar{v_{j}}\;\mathrm{have}\;\mathrm{no}\;\mathrm{side}\;\mathrm{rights} \end{array} \end{array}\right.\begin{array}{cc} & i,j=1,\ldots ,\bar{n} \end{array}$$

where $\bar{V}$ denotes a node, $\bar{E}$ denotes an edge, $\bar{W}$ denotes an edge weight, $\bar{n}$ is the number of nodes in the network, and $\bar{\omega_{ij}}$ denotes the edge weight between node i and node j in $\bar{G}$.

### 2.6. Detecting Community Structure by Using the C-Louvain Algorithm

The Louvain algorithm, introduced by Blondel et al. [13], operates recursively with a two-stage mechanism. Initially, each network node is assigned to its own community. Nodes are then iteratively merged to maximize modularity until no further increase is possible, halting the merging process. Subsequently, the algorithm treats the resulting communities as super-nodes, with inter-community connections serving as edges. It then repeats the first stage process until no further modularity gain is achieved. Accounting for both node movement and community merging, the algorithm’s time complexity is O(n log n).

The Louvain community detection algorithm is the most prevalent for modularity optimization [21]. It is noted for its low computational complexity and high accuracy, effectively identifying community structures in a network [22,23,24,25]. While effective for connected networks, the algorithm struggles with disconnected networks. In such cases, modularity optimization performs global calculations on the entire network’s adjacency matrix, leading to distorted results, unstable iterations, and unreliable community detection [26,27].

To address the aforementioned issues, the C-Louvain algorithm, which integrates connected components with the Louvain algorithm, is developed for community detection within disconnected networks. This enables the effective capture of the structural layout of the network and an improvement in the stability of the detection results.

The C-Louvain algorithm assigns community membership to nodes by identifying connected components, traversing each node within these components, and computing the modularity gain when a node shifts from its current community to that of a neighboring node. To enhance result stability, the algorithm sorts connected components by size and processes nodes by degree to maximize modularity and modularity gain. The modularity and modularity gain are calculated as follows:

Modularity serves as a key metric for identifying community structures within networks. Newman and Girvan (2004) [28] introduced modularity to assess the effectiveness of partitioning a network into communities. The modularity Q is calculated as

(3) $$Q=\frac{1}{2b}\sum_{ij}\left(a_{ij}-\frac{k_{i}k_{j}}{2b}\right)\delta \left(c_{i},c_{j}\right)$$

where *ɑ_ij_* denotes the adjacency matrix of the connected component network; *b* is the number of edges in the connected component network; *k_i_* and *k_j_* are the degrees of node *i* and node *j* in the network, respectively; *k_i_k_j_*/(2*b*) is the expected number of edges between node *i* and node *j*; and *δ(c_i_,c_j_)* takes 1 when nodes *i* and *j* belong to the same cluster, and 0 when the two nodes are located in different clusters.

Modularity gain refers to the change in community modularity when a node is transferred from its original community to an adjacent one [13]. The modularity gain is calculated as

(4) $$\Delta Q=\left[\frac{\sum_{in}+k_{i,in}}{2b}-{\left(\frac{\sum_{tot}+k_{i}}{2b}\right)}^{2}\right]-\left[\frac{\sum_{in}}{2b}-{\left(\frac{\sum_{tot}}{2b}\right)}^{2}-{\left(\frac{k_{i}}{2b}\right)}^{2}\right]$$

where $\sum_{in}$ is the sum of the weights of the connected edges within community A, $\sum_{tot}$ is the sum of the weights of the connected edges involved in the nodes in community A, *k_i_* is the sum of the weights of the connected edges involved in node *i*, and *k_i,in_* is the sum of the weights of the connected edges from *i* to the nodes in A.

**Figure 4:**
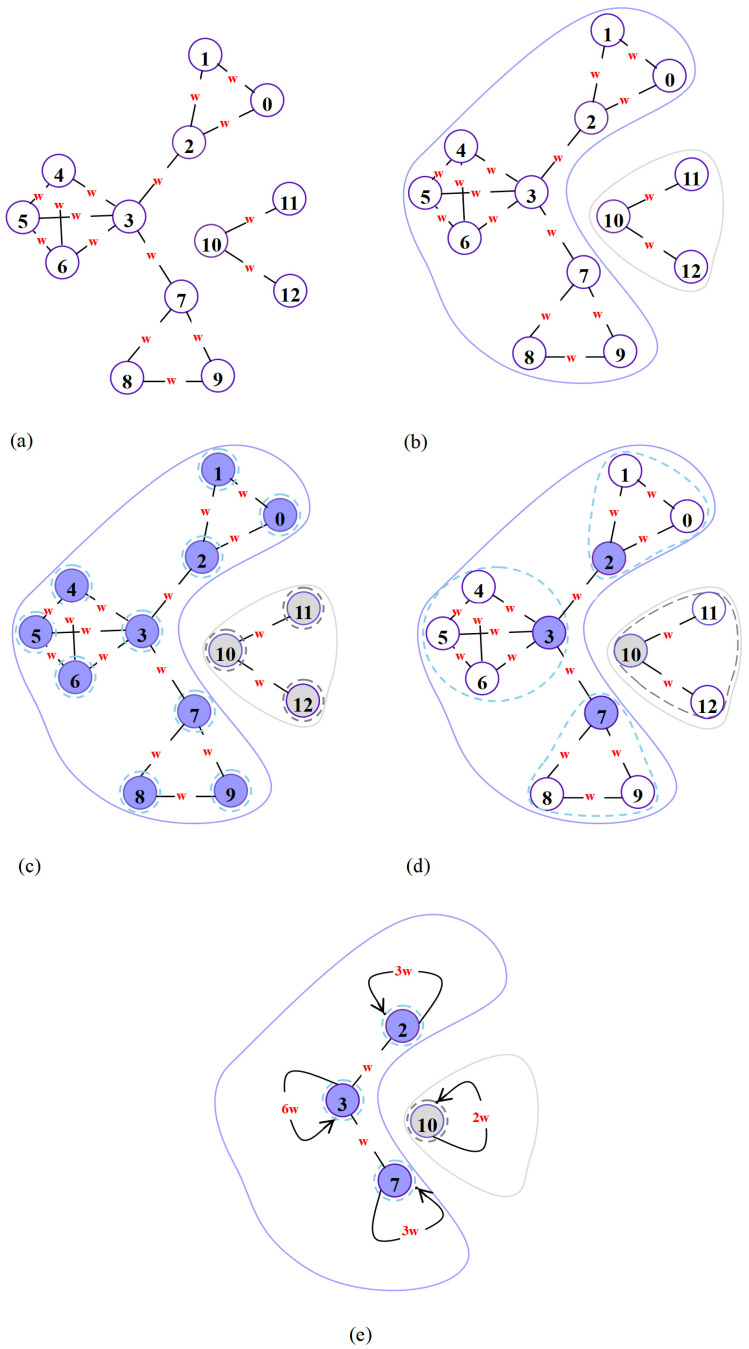
Step-by-step diagram of the C-Louvain algorithm. (**a**) Original network structure. (**b**) Network connectivity component detection. (**c**) Post-initialization network structure. (**d**) First iteration step. (**e**) Second iteration step.

Figure 4 illustrates the C-Louvain community detection algorithm. (a) is the original network structure. (b) shows the identification of connected components. (c) is the network structure after initialization, where the connected components are sorted from largest to smallest, and each node is initially assigned to an independent community. (d) details the first iteration, where nodes within each connected component are assessed based on degree, calculating the change in modularity gain when a node joins its neighbors’ communities. (e) describes the second iteration, where communities formed in the first iteration are condensed into super-nodes. The total edge weight within a community becomes the self-loop weight of the new node, transforming the initial community detection into a new network. The overall modularity of this new network is then evaluated. If the increase in modularity is negligible or absent, the algorithm terminates. Otherwise, this newly generated network undergoes another round of connected component detection and iteration. During each iteration of the C-Louvain algorithm, all nodes in the connected component are traversed to compute the modularity increment for each node. The complexity of a single iteration is O($\bar{n}$). The overall time complexity, considering the number of iterations, is generally O($\bar{n}$ log$\bar{n}$), where log $\bar{n}$ represents the maximum number of iterations. Algorithm 1 illustrates the C-Louvain algorithm process.
**Algorithm 1**: C-Louvain AlgorithmInput: G = (V, E, W): graph representation. Output: C: community sets at each level; Q: modularity at each level. Var: ĉ: vertex u’s best candidate community set. 1 Identify network G connectivity, if it is not connected, find the connected components $\bar{G}=\left(\bar{V},\bar{E},\bar{W}\right)$, and community detection. Sorting by node degree within the connected component. 2 Loop outer
$\begin{array}{l} 3\;C\leftarrow \left\{\left\{u\right\}\right\},\forall u\in \bar{V};\\ 4\;\sum_{in}^{c}\leftarrow \sum {\bar{w}}_{u,v},e\left(u,v\right)\in \bar{E},u\in c\;and\;v\in c;\\ 5\;\sum_{tot}^{c}\leftarrow \sum {\bar{w}}_{u,v},e\left(u,v\right)\in \bar{E},u\in c\;or\;v\in c; \end{array}$
$\begin{array}{l} 6\;\;\;\;Loop\;inner\\ 7\;\;\;\mathrm{for}\;u\in \bar{V}\;and\;u\in c\;do\\ 8\;\;\;\;\hat{c}\leftarrow \;\;\arg\max\;\;\Delta Q_{u\to c^{\prime }};\\ 9\;\;\;\;\forall c^{\prime },\exists e\left(u,v\right)\in \bar{E},v\in c^{\prime }\\ 10\;\;\;\;\;if\;\Delta Q_{u\to \hat{c}}>0\;then\\ 11\;\;\;\;\;\sum_{tot}^{\hat{c}}\leftarrow \sum_{tot}^{\hat{c}}+\bar{w}\left(u\right);\sum_{in}^{\hat{c}}\leftarrow \sum_{in}^{\hat{c}}+{\bar{w}}_{u\to \hat{c}};\\ 12\;\;\;\;\;\sum_{tot}^{c}\leftarrow \sum_{tot}^{c}-\bar{w}\left(u\right);\sum_{in}^{c}\leftarrow \sum_{in}^{c}-{\bar{w}}_{u\to c};\\ 13\;\;\;\;\;\;\hat{c}\leftarrow \hat{c}\cup \left\{u\right\};c\leftarrow c-\left\{u\right\};\\ 14\;\;\;if\;No\;vertex\;moves\;to\;a\;new\;community\;then\\ \;\;\;\;\;\;exit\;inner\;Loop; \end{array}$
$\begin{array}{l} 15\;Q\leftarrow 0;\\ 16\;\mathrm{for}c\in C\;do\\ 17\;\;\;Q\leftarrow Q+\frac{\sum_{in}^{c}}{2b}-{\left(\frac{\sum_{tot}^{c}}{2b}\right)}^{2};\\ 18\;C^{\prime }\leftarrow \left\{c\right\},\forall c\in C;\mathrm{print}C^{\prime }\mathrm{and}Q;\\ 19\;V^{\prime }\leftarrow C^{\prime };\\ 20\;E^{\prime }\leftarrow \left\{e\left(c,c^{\prime }\right)\right\},\exists e\left(u,v\right)\in \bar{E},u\in c,v\in c^{\prime };\\ 21\;{\bar{w}}_{c,c^{\prime }}\leftarrow \sum {\bar{w}}_{u,v},\forall e\left(u,v\right)\in \bar{E},u\in c,v\in c^{\prime };\\ 22\;if\;No\;community\;changes\;then\;exit\;outer\;Loop;\\ 23\;\bar{V}\leftarrow V^{\prime };\bar{E}\leftarrow E^{\prime } \end{array}$Output: C, *Q*

### 2.7. Identifying Key Communities by Using DFS

Depth-first search (DFS) is a graph traversal method named for traversing the nodes of a given graph in a depth-first order. The algorithm selects any node as the root node and traverses other nodes along the path from the root node until no more nodes can be visited, thereby searching for the data of the next node in the graph. If there are still unvisited nodes, the DFS algorithm returns to the node where there is another path to follow. It repeats this process until all nodes have been visited. When there are multiple paths, the criterion for choosing a path is to select the path with the shortest distance from the current node to the nodes on that path [29,30].

Based on the principle of the DFS algorithm, it can be used to find key nodes in a graph. For example, if node u is the parent node of another node v in the graph, and node u is a key node, it must satisfy one of the following two conditions: u is the root node and has at least two child nodes; or u is not the root node and there exists a child node v such that in the subgraph with v as the root, no community has an edge pointing to the DFS graph’s upper-level node of the current community. Combined with the C-Louvain community detection algorithm, the steps for the DFS algorithm to find key communities within a connected component are as follows:

(1) Select any community within the connected component to start the traversal and record its parent community, and check if it is a root community.
(2) If the currently visited community is a root community and has more than two child communities, it is a key community.
(3) If it is not a root community and has a child community, and in the subgraph with the child community as the root, no community has an edge pointing to the DFS graph’s upper-level node of the current community, then it is a key community.
(4) Repeat steps (2)–(3) until no communities can be visited.

### 2.8. Evaluating Spatial Layout Equilibrium Using the Gini Coefficient

Recently, scholars have quantitatively assessed the transportation network equilibrium (including road, bus, and subway networks) using the Lorenz curve and Gini coefficient [31,32,33,34]. The Gini coefficient’s well-established theoretical foundation and grading standards make it advantageous for evaluating equilibrium [35]. To further investigate network layout characteristics, researchers integrate spatial information embedding and topological modeling with complex network theory. By examining the topological structure, they assess the transportation network’s supply–demand equilibrium through correlations between network density and factors like urban form, income, and population density [36,37].

**Figure 5:**
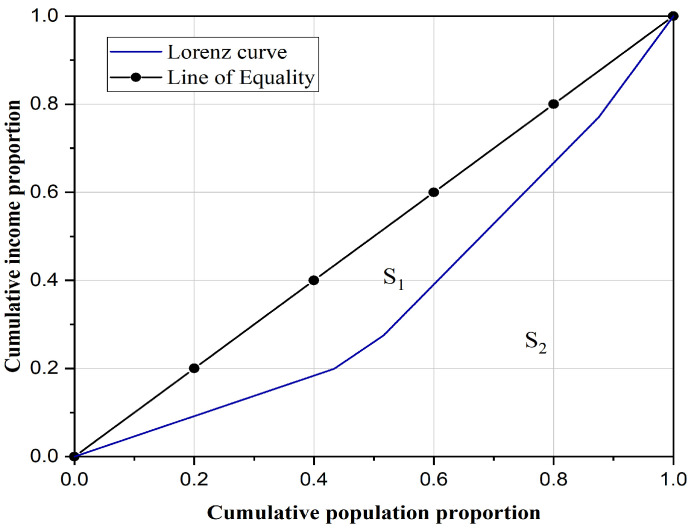
Example of Lorenz curve.

The Gini coefficient is derived from the Lorenz curve, a representation of cumulative frequency distribution. Using residents’ income as an example, the Lorenz curve plots the cumulative population percentage on the x-axis against the cumulative income percentage on the y-axis. Individual incomes are arranged in ascending order and accumulated sequentially to form this curve. The Lorenz curve, alongside the lines of absolute equality and inequality, is illustrated in Figure 5. The Gini coefficient is then calculated as shown in Equation (5).

(5) $$g(x)=\frac{S_{1}}{S_{1}+S_{2}}=\frac{0.5-S_{2}}{0.5}=1-2S_{2}$$

where x is the formal parameter, corresponding to the residents’ income; *g*(x) is the Gini coefficient of indicator x; *S*_1_ is the area enclosed by the Lorenz curve and the line of absolute equality; and *S*_2_ is the area enclosed by the Lorenz curve and the line of absolute inequality.

The function g(x)∈[0, 1] quantifies the deviation of the current distribution of the research object from a state of absolute equality. Higher values indicate a greater imbalance. In the international Gini coefficient evaluation standard, a Gini coefficient greater than 0.4 indicates an imbalanced distribution [38,39].

## 3. Results

### 3.1. Highway Network Modeling

#### 3.1.1. Shenyang Highway Network Modeling

This study models the highway network within the designated area using the igraph library in Python to process highway edge list data and construct a spatial node-based network, defined as G = (V, E, W). Here, V denotes nodes and endpoints of highway sections, E represents the highway sections, and W indicates the length of these sections. The network contains 7177 nodes and 7807 edges, and the maximum node degree is 6. The model visualizes the highway spatial connection relationship, geographic location, and distribution sparsity, and fully covers the actual highway network.

Degree is defined as the number of network edges directly connected to this node, and is usually denoted by *k*. Degree distribution is the most fundamental and important statistical quantity for measuring the structural characteristics of a network, and is usually represented by the distribution function *P_k_*. The degree distribution represents the probability that a randomly selected node in the network has a degree of *k*, that is $p_{k}=N_{k}/N$, where *N_k_* is the number of nodes with a degree of *k*, and *N* is the total number of nodes in the network.

**Figure 6:**
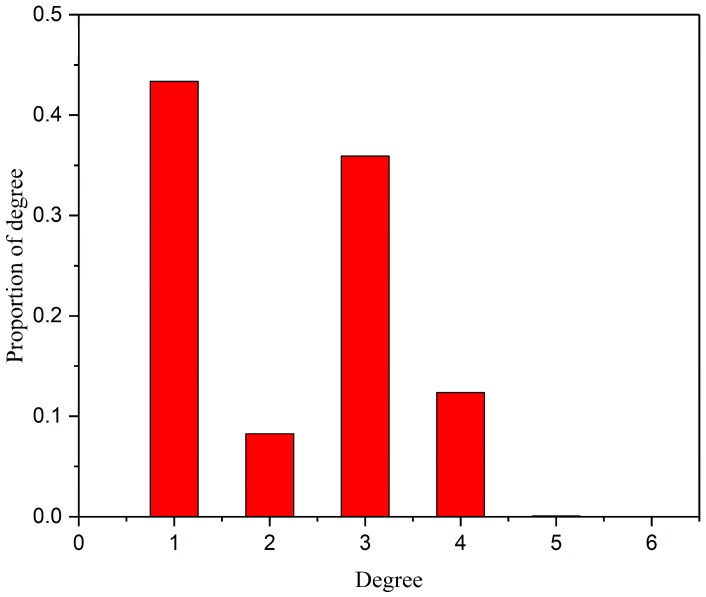
Degree distribution of the highway network.

The degree distribution is a crucial metric for assessing a network’s structural properties. It often adheres to either a Poisson or power-law distribution. In a Poisson distribution, degree values cluster around the average, indicating uniformity, as most nodes have similar degrees near the network’s average. Conversely, a power-law distribution exhibits significant variation, with a few nodes having high degrees and most having low degrees, highlighting network heterogeneity [40,41]. Figure 6 illustrates the degree distribution of Shenyang’s highway transportation network. The degree distribution of the network is not a Poisson distribution; it suggests an imbalanced distribution of node connectivity.

#### 3.1.2. Network Connectivity Analysis

**Figure 7:**
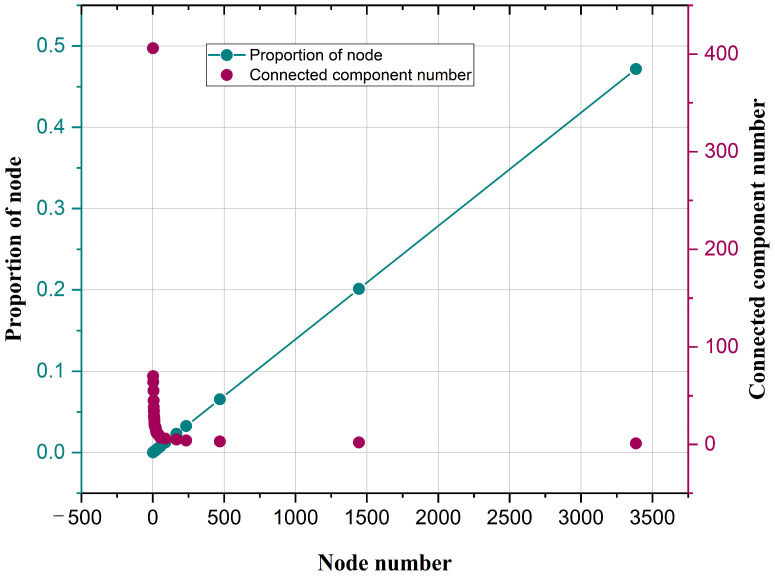
Percentage of connected component nodes.

The Shenyang highway network is a non-connected network. Figure 7 shows the component connectivity node share graph of the Shenyang highway network for the double coordinate axes. It indicates the relationship between the number of connectivity component nodes and proportion of the node, which can be seen as a linear relationship. The maximum number of nodes of the connectivity components is 3385, while the proportion of the node is 0.4717. The connectivity components contain fewer than 10 nodes and account for a higher proportion, indicating that the highway network connectivity is low.

### 3.2. Community Detection Results

**Figure 8:**
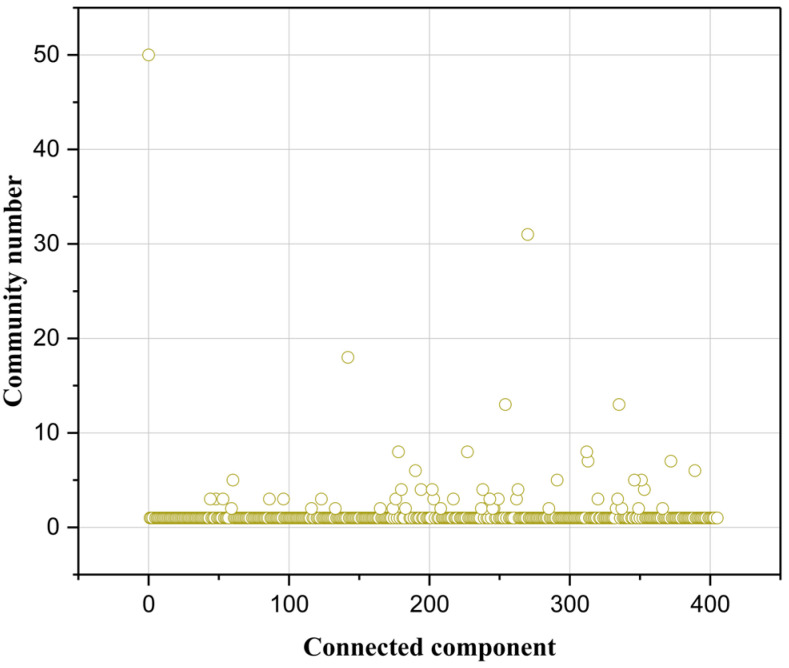
Number of communities.

In this study, the C-Louvain algorithm was applied to detect community structure. Figure 8 represents the number of communities within each connectivity component. The maximum connectivity component is divided into 50 communities. There are five connectivity components that contain more than ten communities, and most of the connectivity components contain a single community. It indicates that the local highway network has heterogeneity in its structure, with multiple disconnected communities.

### 3.3. Key Community Identification

#### 3.3.1. Community Structure Network Analysis

**Figure 9:**
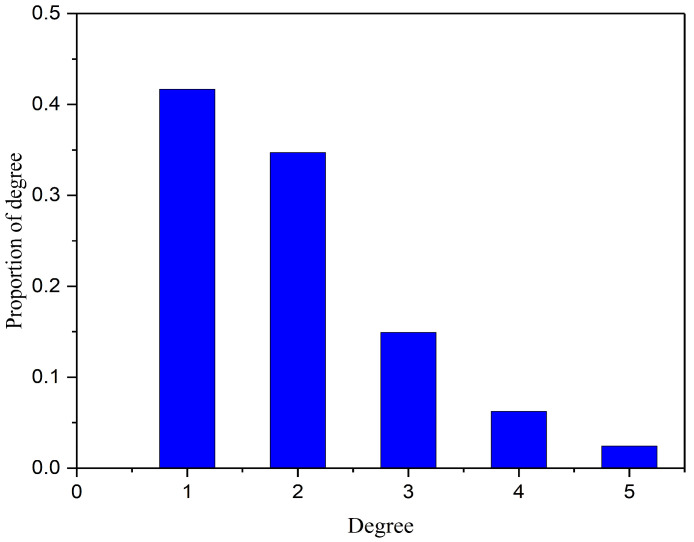
Community network degree distribution.

From the results of the community detection, in order to visualize the size of the communities and the connection between the communities, the community structure of the Shenyang highway network was constructed. The maximum node degree of the network is 5, and the average degree is 1.9863. It can be seen that the distributions of the large and small communities differ greatly. There is a small amount of highway connection between large communities, and most of the small communities show no highway connection. This indicates that the communities are relatively independent of each other. The highway connectivity within the communities is good, but the highways outside the communities are basically unconnected. There is overlap and intersection among communities, indicating that the highway network breaks through administrative divisions and achieves shared functionality. In traffic network planning, different levels of highways are often shared in certain sections or areas. The aim is to optimize the network layout by sharing resources and improving overall traffic efficiency. Figure 9 shows the community network degree distribution. This indicates that the community network is non-uniform, with an imbalanced distribution of inter-community connections.

#### 3.3.2. Key Community Identification Results

A community is considered key if its removal increases the number of connected components in a graph, indicating its bridging role and impact on network connectivity, reliability, and stability. Identifying and analyzing such key communities can provide insights into the supply–demand balance mechanism of the entire network.

**Figure 10:**
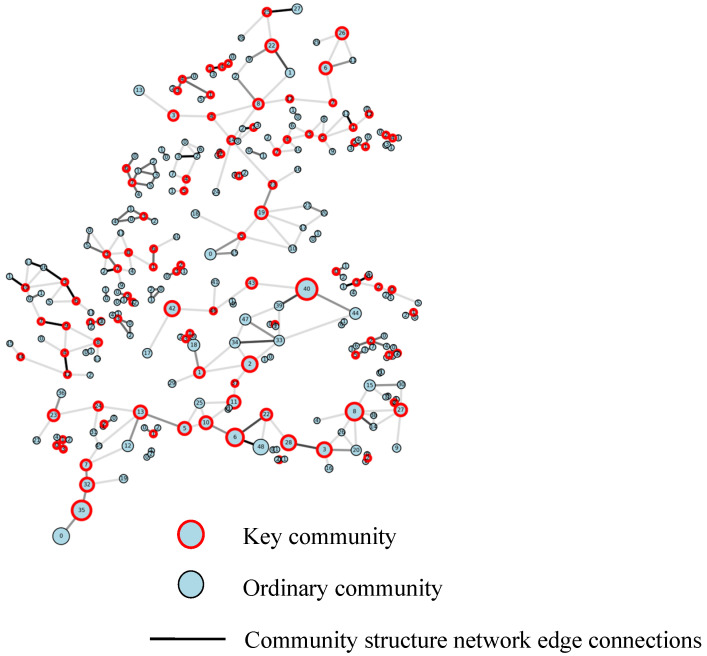
Key community detection.

Utilizing the DFS algorithm and the community structure network diagram, we identified key communities within each connected component, as shown in Figure 10, where the red circles indicate the key communities in the study area. These larger key communities predominantly cluster around the urban area, indicating a spatial layout bias in the network. The detection of key communities within the highway community network reveals significant differences, underscoring the stability of the network formed by varied highway grades and the critical structural roles of traffic connections between urban and rural areas.

### 3.4. Gini Coefficient Equalization Evaluation Results

Population, GDP, and car ownership serve as proxies for population demand, economic demand, and travel demand, respectively. Highways indicate the supply status of transportation infrastructure. By integrating supply–demand balance analysis with the Lorenz curve, the Gini coefficient is calculated to assess the spatial layout equilibrium of the highway network.

**Figure 11:**
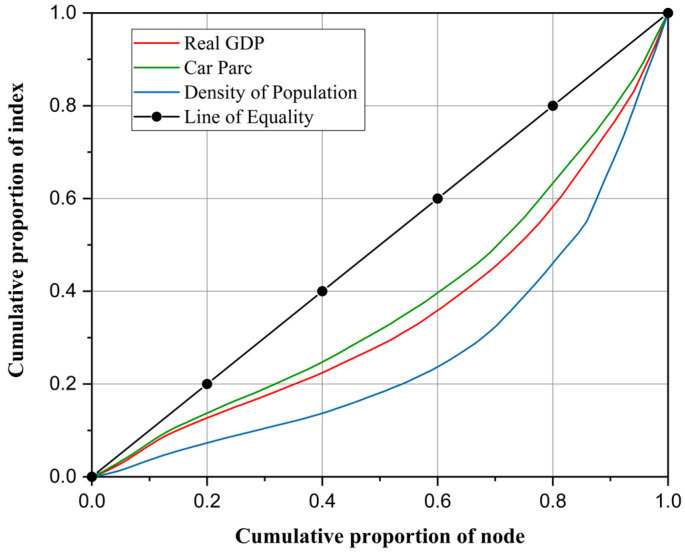
Community-related Lorenz curve.

Interpolation analysis was used to obtain highway node data for three demand indicators in each community, namely population, GDP, and car ownership. The node count within each community indicates highway supply status. The Lorenz curve for the study area is depicted in Figure 11. The population Gini coefficient is 0.4622, the GDP Gini coefficient is 0.2996, and the car ownership Gini coefficient is 0.2481. This indicates that the level of economic development in each community and the travel demand match the supply of the highway network, whereas the population distribution is more mismatched with the supply of the highway network. From the county Gini coefficient analysis, the regional highway supply in each county does not match the regional population demand and economic demand, but matches the travel demand more. By breaking through the county-level administrative boundaries and considering the spatial structure grouping characteristics of the highway network itself, the community structure is able to analyze the matching relationship between the supply and demand of highway groups, reflecting the degree of highway spatial layout imbalance.

**Figure 12:**
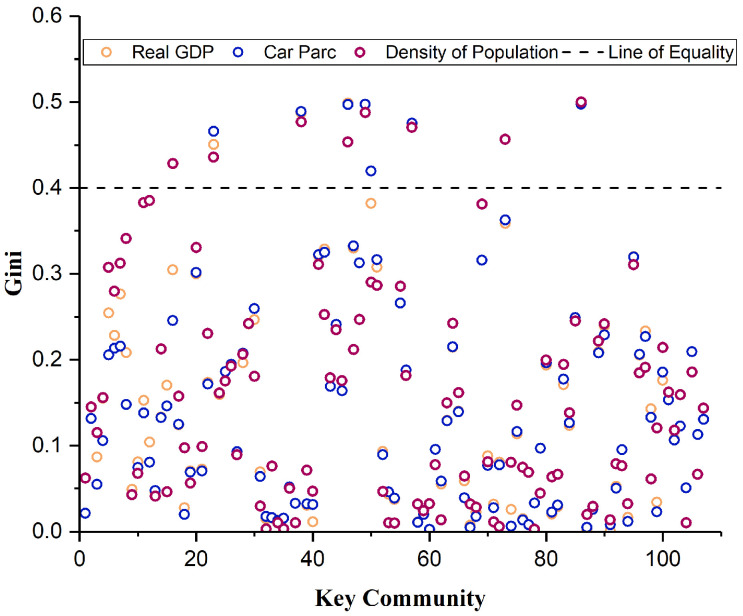
Gini coefficients by indicator for key communities.

**Figure 13:**
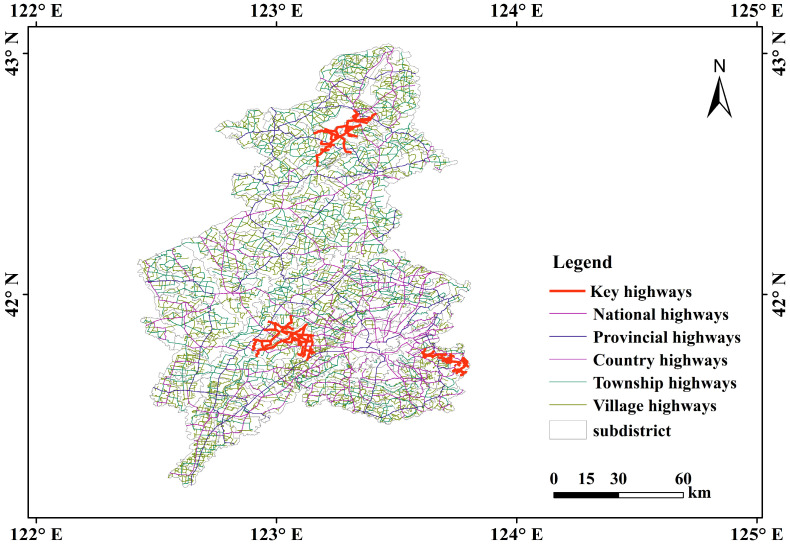
Imbalanced spatial layout of the highway network in Shenyang.

Examine the supply–demand balance of key communities to identify imbalances. Figure 12 shows the key communities’ Gini coefficients. The number of communities with a population Gini coefficient greater than 0.4 is eight communities, the number of communities with a GDP Gini coefficient greater than 0.4 is six communities, and the number of communities with a car ownership Gini coefficient greater than 0.4 is seven communities. The number of communities with all three indices greater than 0.4 is six, which means that they are imbalanced communities. The spatial layout of the highway network in the imbalanced communities expresses the imbalanced spatial layout of the highway network in the study area, as shown in Figure 13. The areas are mainly located in Kangping County in the north of Shenyang, Tiexi District in the southwest, and Hunnan District in the southeast. The proportion of county highway mileage is 7.07%, that of township highway mileage is 49.40%, and that of village highway mileage is 43.53%, mainly concentrated on township highways and village highways.

### 3.5. Algorithm Comparison

The advancement of modern graph theory techniques has significantly influenced the study of complex networks. Researchers frequently employ graph theory to identify community structures through several methods: (1) graph partitioning, exemplified by the Girvan–Newman (GN) algorithm [42]; (2) modularity optimization, including the Fast Newman (FN) [43], Louvain [13], and Leiden algorithms [44]; (3) label propagation, demonstrated in the label propagation algorithm (LPA) [45], hub-based algorithm [46], and COPRA algorithm [47]; and (4) dynamical methods, like the finding and extracting communities (FEC) [48], INFOMAP [49], and Ronhovde–Nussinov (RN) algorithms [50]. Of these, modularity optimization is the most prevalent approach for detecting community structures.

This study assesses the C-Louvain algorithm’s effectiveness through comparative experiments with modularity-based community detection algorithms, specifically the FN, Leiden, and Louvain algorithms. We employ three indicators: modularity Q, coefficient of variation CV, and time complexity O, to evaluate the algorithms’ accuracy, volatility, and efficiency [51]. Modularity Q, an indicator of community division quality, reflects accuracy; higher values denote superior community division. The coefficient of variation CV, defined as σx/Ex (where σx is the standard deviation and Ex the expected value), standardizes data dispersion, indicating modularity fluctuation. A lower CV suggests less dispersion, implying higher data concentration and stability, thereby reflecting each algorithm’s randomness and reliability. Time complexity measures how an algorithm’s execution time scales with input size, assessing its efficiency.

To mitigate the variability inherent in single experimental tests, this study conducts repeated experiments on the highway transportation network in Shenyang, averaging the results for comparison, as detailed in Table 2. The findings demonstrate that the proposed method excels in modularity, the coefficient of variation, and time complexity. As network complexity increases, the modularity value nears 1, indicating enhanced community division quality; the coefficient of variation declines, reflecting more reliable detection outcomes; and the time complexity is logarithmic, underscoring the algorithm’s efficiency. These results confirm that the proposed method significantly enhances the quality and stability of community division.

## 4. Discussion

### 4.1. Algorithm Discussion

To reveal the local structural characteristics of the network, the community detection method is introduced to describe the structure of the highway network more precisely, providing structured input for the analysis of spatial layout equilibrium. The Louvain algorithm [13], due to its inability to handle disconnected networks, has a problem of distorted optimization of modularity calculation results and is thus unable to obtain reliable community detection results. In this paper, we introduce a new algorithm, the C-Louvain algorithm, which is the first to tackle improving the stability of detection results of unconnected networks by calculating the modularity and modularity gain of node degree ordering of connected components.

The Louvain algorithm can yield non-positive modularity values when applied to disconnected networks, leading to meaningless community mergers and failing to accurately represent community divisions within connected components. Additionally, the algorithm’s results are susceptible to the randomness of node processing order, necessitating enhanced stability. The C-Louvain algorithm addresses these issues by detecting connected components and evaluating each node within them. It calculates the modularity gain when a node is moved from its current community to that of its neighboring nodes, thereby determining community affiliation. During this process, connected components are prioritized by size, and nodes within these components are processed according to their degree to maximize modularity and modularity gain. Through algorithm comparison, it can be concluded that this method significantly improves the accuracy, stability and efficiency of community detection.

### 4.2. Traffic Policy Implication

Quantitative analysis of the spatial layout equilibrium of the highway transportation network reveals significant imbalances in the spatial layout of highway networks in different regions. Several key corridors and urban nodes exhibit persistent supply shortages, while the infrastructure utilization in some surrounding areas is relatively low, indicating a mismatch between construction investment and actual demand. These findings reveal the dual challenges of efficiency and equity in highway network layouts, with policy implications that go far beyond infrastructure itself and touch the heart of regional development.

(1) The policy should shift from “efficiency first” to “efficiency and equity equally.” While investment in high-demand corridors is important, attention must be paid to areas marginalized by inadequate infrastructure. Policymakers should introduce a framework for assessing transport equity to ensure that people in different income groups and in urban, rural, and remote areas enjoy basic mobility rights. This means that projects that improve “service gaps” should be given higher weight in investment decisions to avoid transport networks becoming tools that exacerbate social stratification.
(2) Equilibrium should be used as a lever to achieve sustainable development goals. Areas with low infrastructure utilization are often accompanied by extensive land development and ecological pressure. Through equilibrium analysis, investment can be guided to stock optimization rather than incremental expansion. It can not only improve efficiency but also reduce the occupation of land and environment. At the same time, optimizing high-demand corridors can reduce congestion and carbon emissions across the entire road network, directly contributing to the “double carbon” goal.
(3) A resilient regional economy should be built through balanced planning. A balanced network implies multiple growth poles and connecting channels within a region, rather than a single central dependency. This multicentric, networked structure exhibits greater resilience in the face of external shocks. Policies should encourage the cultivation of characteristic industrial nodes in surrounding areas and connect them with core cities through efficient road networks to form regional economies with complementary functions and coordinated development, thus supporting long-term and sustainable regional integration.

In conclusion, equilibrium analysis is not only a technical tool, but also a policy philosophy. It requires decision-makers to take into account social equity and environmental responsibility while pursuing economic benefits, so as to ensure that transportation development truly serves the all-round development of people and the sustainable prosperity of the region.

## 5. Conclusions

This study aims to explore the application of complex network community detection methods for quantitative analysis of the layout equilibrium of the highway network. Through the analysis of real network data, the conclusions are as follows:

(1) There are significant differences in the number of communities within different connected components, which reflects the heterogeneity of the network’s structure.
(2) Using the Gini coefficient to quantitatively evaluate the supply–demand balance of key communities, the results show that in the study area, the proportion of county highway mileage is 7.07%, that of township highway mileage is 49.40%, and that of village highway mileage is 43.53%, mainly concentrated on township highways and village highways.
(3) The originality of this work lies in its integration of complex network theory with transportation engineering, providing both methodological innovation and practical implications for urban planning. Community detection has opened up new avenues for research in traffic management systems.
(4) Future work should involve on-site verification to enhance expert knowledge and validate the accuracy of the constructed network data. Results may differ when employing other community detection algorithms, necessitating an analysis of various influencing factors to achieve more precise community detection outcomes. We will study the advantages and disadvantages of the C-Louvain method in comparison with the hybrid methods or multi-criteria equilibrium analysis. We will also study dynamic or temporal analyses of the adaptability of the research methods. Concurrently, the influence of interpolating socioeconomic indicators into the research results is analyzed. To enhance the versatility and real-world applicability of C-Louvain, studies should focus on conducting case studies in multiple cities with diverse geographic and socioeconomic contexts to evaluate the transferability of C-Louvain. Additional data sources should be integrated to enrich the multimodal network representation and improve the accuracy of community detection.

## Figures and Tables

**Table 1:** Data collection sections.

Highway Class	Number of Highways	Length (km)
National highways	8	799.33
Provincial highways	14	1058.28
County highways	86	1427.02
Township highways	442	3382.83
Village highways	2070	5853.09

**Table 2:** Comparison of community detection algorithms.

Model	Q	CV	O
FN	0.9734	3.4216	O(n2 log n)
Louvain	0.9537	0.0006	O(n log n)
Leiden	0.9561	0.4374	O(n log n)
C-Louvain	0.9739	0.0003	$O(\bar{n}$$\;\log\bar{n}$)

## Data Availability

National highway, provincial highway, and county highway data were sourced from OpenStreetMap (OSM) 2020 highway data. Township and village highway data can be obtained on request by e-mail from https://ciigis.lntu.edu.cn/ (accessed on 25 October 2023).
